# Aryl Hydrocarbon Receptor (AhR) Limits the Inflammatory Responses in Human Lung Adenocarcinoma A549 Cells via Interference with NF-κB Signaling

**DOI:** 10.3390/cells11040707

**Published:** 2022-02-17

**Authors:** Gerardo Vázquez-Gómez, Martina Karasová, Zuzana Tylichová, Markéta Kabátková, Aleš Hampl, Jason Matthews, Jiří Neča, Miroslav Ciganek, Miroslav Machala, Jan Vondráček

**Affiliations:** 1 Department of Cytokinetics, Institute of Biophysics of the Czech Academy of Sciences, 61265 Brno, Czech Republic; karasova@ibp.cz (M.K.); tylichova@ibp.cz (Z.T.); kabatkova@ibp.cz (M.K.); 2 Department of Experimental Biology, Faculty of Science, Masaryk University, 62500 Brno, Czech Republic; 3 Department of Histology and Embryology, Faculty of Medicine, Masaryk University, 62500 Brno, Czech Republic; ahampl@med.muni.cz; 4 International Clinical Research Center, St. Anne’s University Hospital Brno, 65691 Brno, Czech Republic; 5 Department of Nutrition, Institute of Basic Medical Sciences, University of Oslo, 0372 Oslo, Norway; jason.matthews@medisin.uio.no; 6 Department of Pharmacology and Toxicology, University of Toronto, 1 King’s College Circle, Toronto, ON M5S 1A8, Canada; 7 Department of Pharmacology and Toxicology, Veterinary Research Institute, 62100 Brno, Czech Republic; jir.neca@gmail.com (J.N.); ciganek@vri.cz (M.C.); machala@vri.cz (M.M.)

**Keywords:** AhR, inflammation, alveolar epithelial type II cells, NF-κB, prostaglandins, cytokines

## Abstract

Apart from its role in the metabolism of carcinogens, the aryl hydrocarbon receptor (AhR) has been suggested to be involved in the control of inflammatory responses within the respiratory tract. However, the mechanisms responsible for this are only partially known. In this study, we used A549 cell line, as a human model of lung alveolar type II (ATII)-like cells, to study the functional role of the AhR in control of inflammatory responses. Using IL-1β as an inflammation inducer, we found that the induction of cyclooxygenase-2 and secretion of prostaglandins, as well as expression and release of pro-inflammatory cytokines, were significantly higher in the AhR-deficient A549 cells. This was linked with an increased nuclear factor-κB (NF-κB) activity, and significantly enhanced phosphorylation of its regulators, IKKα/β, and their target IκBα, in the AhR-deficient A549 cells. In line with this, when we mimicked the exposure to a complex mixture of airborne pollutants, using an organic extract of reference diesel exhaust particle mixture, an exacerbated inflammatory response was observed in the AhR-deficient cells, as compared with wild-type A549 cells. Together, the present results indicate that the AhR may act as a negative regulator of the inflammatory response in the A549 model, via a direct modulation of NF-κB signaling. Its role(s) in the control of inflammation within the lung alveoli exposed to airborne pollutants, especially those which simultaneously activate the AhR, thus deserve further attention.

## 1. Introduction

The lung is continuously exposed to airborne environmental agents present in the atmosphere and its epithelial cell population plays a major role in recognizing potential stressors [1], thus contributing to the maintenance of lung homeostasis [2]. The alveolar epithelium consists primarily of two types of alveolar epithelial cells: type I cells (ATI) and type II cells (ATII). ATI cells are involved primarily in gas exchange and maintenance of fluid balance [3]. The ATII cells are a major source of lung surfactant, they act as a progenitor cell population crucial for alveolar epithelium renewal, and they contribute to regulation of the immune/inflammatory responses within alveoli [4,5,6,7,8].

The aryl hydrocarbon receptor (AhR) is a highly conserved ligand-activated transcription factor that is expressed in most human tissues, including alveolar epithelium [9]. The AhR is well known for its control of the metabolism and bioactivation of airborne pollutants, including polycyclic aromatic hydrocarbons (PAHs). In the canonical AhR signaling pathway, following its ligand binding, the AhR translocates to the nucleus, where it heterodimerizes with the AhR nuclear translocator, and binds to enhancer sequences within regulatory regions of target genes, including not only those encoding for phase I and phase II metabolizing enzymes, but also a number of genes involved in important physiological processes [10]. There is growing evidence that the cellular functions of the AhR extend beyond xenobiotic metabolism, and that this transcription factor is an important regulator of immune system and inflammatory responses [10,11]. Experimental evidence suggests that the AhR exerts its immunomodulatory roles via cross-talk with different signaling pathways in a tissue-dependent manner [12,13,14,15,16,17,18,19,20,21]. AhR-deficient mice exhibit significantly greater neutrophilia than wild-type mice after cigarette smoke exposure, which was suggested to be due to the AhR acting via a non-canonical protein interaction with the nuclear factor-κB (NF-κB) subunits [13]. The AhR has been reported to be involved in the attenuation of inflammation by decreasing the expression of prostaglandin-endoperoxide synthase 2/cyclooxygenase-2 (COX-2), the principal enzyme involved in production of pro-inflammatory prostaglandins (PGs), via the nuclear sequestration of an RNA-binding protein that functions to stabilize COX-2 mRNA, HuR [20]. In addition, the unliganded AhR may associate with the NF-κB protein RelA in human lung cells, thus modulating the NF-κB activity and upregulating interleukin-6 (IL-6) expression [15]. AhR activation may also promote RelA/p65 protein degradation through both the ubiquitin-proteasome system and via lysosomal degradation [14]. The heightened inflammatory response observed in the AhR^−/−^ fibroblasts could be also coupled to the loss of the NF-κB family member RelB in the AhR^−/−^ fibroblasts [12]. A recent study has also reported that anti-inflammatory action of the AhR could be independent of its binding to dioxin-responsive elements (DRE) [22]. Together, these data suggest that the AhR might interfere with the NF-κB signaling in respiratory system through multiple mechanisms. However, it is presently not clear if the attenuation of NF-κB activity is relevant for the AhR action also within the context of ATII-like cells.

Taken together, there is limited information about the immunomodulatory role of the AhR in the human alveolar epithelium, as most of the studies have focused on bronchial epithelium or lung fibroblasts. Since alveolar epithelium can be exposed to large quantities of airborne particles that induce inflammatory responses, but also contain the AhR ligands, including PAHs and various PAH derivatives [23,24,25], it is important to better understand the mechanisms underlying the cross-talk of the AhR signaling and inflammatory mediators in the ATII cells. Here, we used a human model of ATII-like cells, lung adenocarcinoma A549 cell line, in order to study the functional role of the AhR in the modulation of inflammatory responses (production of cytokines, chemokines and prostaglandins), and to examine the mechanisms underlying the cross-talk of the AhR with inflammatory signaling.

## 2. Materials and Methods

### 2.1. Chemicals

Human recombinant IL-1β was purchased from Merck (Prague, Czech Republic) and reconstituted according to the supplier’s instructions. Stock solutions were stored at −20 °C. Dimethyl sulfoxide (DMSO) and CH-223191 were obtained from Merck. 2,3,7,8-Tetrachlorodibenzo-*p*-dioxin (TCDD) was purchased from Cambridge Isotope Laboratories (Andover, MA, USA) and dissolved in DMSO. Standard reference mixture of diesel exhaust particles (DEP) produced by a combination of direct injection four-cycle diesel engines SRM 1650b [26] was purchased from the National Institute of Standards and Technology (Gaithersberg, MD, USA). It was extracted and fractionated based on different molecule polarity to separate different classes of polycyclic aromatic compounds, as previously described [27,28]. Both crude SRM1650b extract (CE) and its polar (F3) fraction were used for further analyses, at non-cytotoxic concentration (0.1 mg SRM equivalents/mL), which was selected based on preliminary experiments. All other chemicals were obtained from Merck, if not stated otherwise.

### 2.2. Cell Line

Human lung carcinoma A549 cells were obtained from the American Type Culture Collection (ATCC; Manassas, VA, USA). Cells were maintained in Dulbecco’s modified Eagle’s medium/nutrient mixture F-12 Ham (DMEM/F12, Thermo Fisher Scientific, Waltham, MA, USA) supplemented with antibiotics and 10% fetal bovine serum (FBS; *v*/*v*) (Thermo Fisher Scientific). The cells were cultivated at 37 °C in 5% CO_2_ and 95% humidity, and sub-cultured upon reaching 80% confluence.

### 2.3. Generation of A549 Knock-Out Cells

A CRISPR/Cas9 expression vector encoding green fluorescent protein (pSpCas9(BB)-2A-GFP(PX458; Addgene, Cambridge, MA, USA) was used for generation of the AhR-deficient cells. A549 cells were transfected with a mixture of vector DNA and Lipofectamine 3000 + P3000 (Thermo Fisher Scientific). The selection of GFP-positive cells was completed by FACS sorting (BD Aria II Sorp, Becton Dickinson, Franklin Lakes, NJ, USA). GFP-positive cells were sorted as single cell suspension into 96-well plates. Individual clones were then expanded. DNA was isolated by QIAamp DNA Mini Kit (Qiagen, Hilden, Germany). The functionality of CRISPR/Cas9 system was checked using SURVEYOR mutation detection kit (Integrated DNA Technologies, Skokie, IL, USA). In CRISPR/Cas9-active clones, the levels of the AhR protein were determined by Western blotting using rabbit monoclonal anti-human AhR antibody (Cell Signaling Technology, Danvers, MA, USA; Cat. No. 83200). The AhR knockout was then verified by sequencing. PCR products obtained by PCR amplification of the target sequence were purified using QIAquick PCR Purification Kit (Qiagene) and inserted into plasmid vector (pGEM_T Easy Vector Systems, Promega, Madison, WI, USA), and *E. coli* DH5α (MAX Efficiency DH5α Competent Cells, Thermo Fisher Scientific) were transformed and cultivated. Plasmid DNA was isolated using GeneJET Plasmid Miniprep kit (Thermo Fisher Scientific), and preparation of samples for sequencing was performed by Mix2Seq overnight sequencing kit according to the instructions provided by sequencing services (Eurofins Genomic, Vienna, Austria). The results are shown in Supplementary Figure S1. 

### 2.4. Western Blotting

Cells were seeded in 6-well plates, at density of 30,000 cells per cm^2^ and grown for 24 h before the treatments. All treatments are described in the legends to the Figures. Following the treatments, cell lysates were prepared in whole cell lysis buffer (1% SDS, 10% glycerol, 100 mM Tris pH 7.4, 1 mM NaF, 1 mM Na_3_VO_4_, 1 mM PMSF), sonicated and protein concentration was determined. SDS loading buffer (240 mM Tris–HCl pH 6.8, 6% SDS, 0.02% bromophenol blue, 30% glycerol, 3% β-mercaptoethanol) was added to samples, which were then boiled (10 min). Protein samples (20 μg per lane) were then separated by SDS-PAGE (10% polyacrylamide gels) and blotted to PVDF membranes (GE Healthcare, Little Chalfont, UK) in a buffer containing 192 mM glycine, 25 mM Tris, and 10% methanol. The membranes were blocked in 5% milk in wash buffer (0.05% Tween-20 in 20 mM Tris; pH 7.4; 100 mM NaCl) for 1 h. We used the following primary antibodies: rabbit anti-AhR (Cell Signaling Technology); mouse anti-COX-2 (Santa Cruz Biotechnology, Dallas, TX, USA; Cat. No. sc-19999); rabbit anti-mPGES-1 (Abcam, Cambridge, UK; Cat. No. 62050); rabbit anti-phospho-IKKα/β Ser1176/180 (Cell Signaling Technology, Cat. No. 2697); mouse anti-IKKα (Cell Signaling Technology, Cat. No. 11930); rabbit anti-phospho-IκBα Ser32 (Cell Signaling Technology, Cat. No. 2859); mouse anti-IκBα (Cell Signaling Technology, Cat. No.4814) and mouse anti-β-actin (Merck). All primary antibodies were incubated with the blots overnight (at 4 °C), washed, and then incubated with secondary antibodies coupled to horseradish peroxidase, anti-mouse IgG (GE Healthcare, Chicago, IL, USA; Cat. No. NA931V) or anti-rabbit IgG (GE Healthcare, Cat. No. NA934V), for 1 h. For detection, we used Clarity™ Western ECL Substrate (Bio-Rad, Prague, Czech Republic). The visualization was performed with the ChemiDoc imaging system (Bio-Rad).

### 2.5. Luciferase Gene Reporter Assay

Cells were seeded at a density of 30,000 cells per cm^2^ in 24-well plates in 1 mL of DMEM/F12 medium supplemented with 10% FBS without antibiotics. Cells were then transiently transfected with pNFκB-luc reporter construct (Stratagene, San Diego, CA, USA) and pRL-TK vector encoding *Renilla* luciferase (Promega). Transfections were performed in a final volume of 500 μL of Opti-MEM medium (Thermo Fisher Scientific) with 50 ng/mL of pRL-TK and 200 ng/mL of pNFκB-luc plasmids, and with 1 μL of Lipofectamine 2000 (Thermo Fisher Scientific). After 6 h, the medium was changed to DMEM-F12 (supplemented with antibiotics and 10% FBS), and 24 h later, the treatments were performed. After incubation, cells were lysed in passive lysis buffer (Promega) and luciferase activity was determined with Dual-Luciferase Reporter Assay (Promega), using LM-01T luminometer (Immunotech, Prague, Czech Republic). The *Renilla* luciferase activity was used for normalization of the firefly luciferase activity. 

### 2.6. Real-Time Quantitative RT-PCR (RT-qPCR)

Cells were seeded in 6-well plates at density of 30,000 per cm^2^ in 2 mL of supplemented DMEM/F12 and grown for 24 h before the treatments. All treatments are described in legends to Figures. Total RNA was isolated with High Pure RNA Isolation kit (Roche Diagnostics, Mannheim, Germany; Cat. No. 11828665001). The cDNA was prepared with Transcriptor First Strand cDNA Synthesis Kit (Roche, Cat. No. 04897030001). The quantitative polymerase chain reaction (qPCR) was performed on the Lightcycler 480 II using the LightCycler^®^ 480 Probe Master (Roche Diagnostic Corporation, Prague, Czech Republic). Primers and probes were obtained from Generi Biotech (Hradec Králové, Czech Republic), Roche Diagnostics (Mannheim, Germany) and Thermo Fisher Scientific, and they are listed in Supplementary Table S1. For normalization of gene expression, we used TATA-box binding protein gene (TBP) as a house-keeping gene.

### 2.7. Liquid Chromatography-Tandem Mass Spectrometry (LC-MS/MS)

Cells were seeded in 6-well plates at density of 30,000 cells per cm^2^ and grown for 24 h before the treatments. Following treatments, the cell culture medium was isolated and then stored at −20 °C. The PGs concentration was measured by LC-MS/MS as previously described [29]. Briefly, solid-phase-extraction was used for extraction of eicosanoids from cell culture media, using SELECT HLB SPE 1 mL (30 mg) cartridges (Supelco, Prague, Czech Republic). The samples were then under a stream of nitrogen and dissolved in 60 μL of methanol (aliquots of 5 μL were then used for further analysis). Samples were further analyzed using Agilent 1200 chromatographic system (Agilent Technologies, Waldbronn, Germany), using Ascentis Express C18, 2.1 150 mm, 2.7 μm particle size column (Supelco, Bellefonte, PA, USA). The separation conditions have been described previously [29]. For detection of analytes, we used Agilent 6410 Triple Quad LC/MS (Agilent Technologies) with an electrospray interface (ESI). Its operational conditions have been described previously [29]. Standards of PGE_2_, PGD_2_, PGA_2_, PGF_2α_, 8-iso-PGF_2α_ and other eicosanoids were purchased from the Cayman Chemical Company (Ann Arbor, MI, USA).

### 2.8. Enzyme-Linked Immunosorbent Assays (ELISA) 

Cells were seeded in 6-well plates at density of 30,000 cells per cm^2^ and grown for 24 h before the treatments. All treatments are described in the legends to the Figures. After treatment, the culture medium was aspirated and stored at −20 °C until further analyses. The content of cytokines was measured using Uncoated ELISA kits (Thermo Fisher Scientific), for IL-6, IL-8 and CCL2/MCP1, respectively. Briefly, high-affinity protein binding plates were incubated with capture antibody overnight. The wells were then blocked with PBS supplemented with fetal bovine serum for 1 h. After washing, the plates were incubated with conditioned media for 2 h. Subsequently, wells were incubated with the detection antibody for 1 h. After washing, Streptavidin-HRP was added to all wells and incubated for 30 min. Finally, substrate solution was added, incubated for 15 min and the reaction was stopped. Optical densities were measured at 450 nm.

### 2.9. Statistical Analyses

The data are presented as means + standard deviation (SD) of at least three independent experiments The differences between the groups were assessed using two-way ANOVA, followed by Tukey multiple comparison test, or *t*-test, where appropriate. An associated probability *p*-value of <5% was considered significant.

## 3. Results

### 3.1. Generation of the AhR Knockout Cells by CRISPR/Cas9 

**Figure 1:**
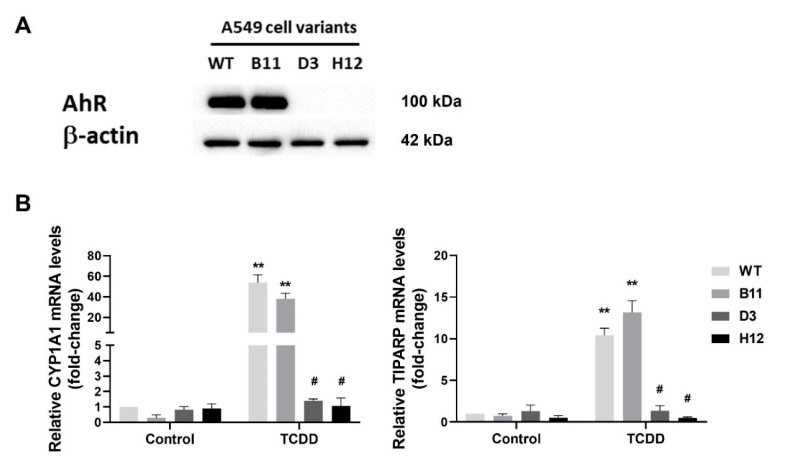
Expression of the AhR and its function are disrupted in the A549 AhR KO cells. (**A**) Whole cell extracts from A549 wild-type cells (WT), A549 cells transfected with a CRISPR/Cas9 empty vector (B11) and the A549 AhR KO cell variants D3 and H12 were analyzed by Western blotting, in order to detect the AhR protein. β-Actin was used as a loading control. The results are representative of two independent analyses; (**B**) A549 WT, A549 B11 and the A549 AhR KO cell variants D3 and H12 were exposed to the AhR agonist TCDD (10 nM) or its diluent (DMSO; 0.01% *v*/*v*), as a control, for 24 h. After incubation, CYP1A1 and TiPARP mRNA levels assessed by RT-qPCR. The data represent means + SD of three independent experiments. ** Denotes significant difference (*p* < 0.01) between the TCDD-treated cells and the respective control group. # Denotes significant difference (*p* < 0.05) between the TCDD-treated AhR KO clones and TCDD-treated WT cells.

To evaluate the contribution of the AhR to the control of inflammatory response in lung ATII-like cells, we prepared A549 The AhR-knockout cell variants (AhR KO) by CRISPR/Cas9 technique. We confirmed that the AhR protein was not detectable by Western blotting in AhR KO clones D3 and H12, as compared with control wild-type cells (WT) and transfection control cells (B11 clone) (Figure 1A). To confirm the disruption of AhR signaling in D3 and H12 cells, we then exposed the cells to the high affinity AhR agonist TCDD, and we determined the induction of the CYP1A1 and TiPARP mRNAs. As shown in Figure 1B, TCDD failed to induce CYP1A1 or TiPARP mRNA in both D3 and H12 clones, whereas their induction was significant in both WT cells and in B11 clone. These results confirmed the absence of a functional AhR signaling pathway in the two AhR KO cell clones, D3 and H12.

### 3.2. AhR Deficiency Increases Production of Prostaglandins in A549 Cells

**Figure 2:**
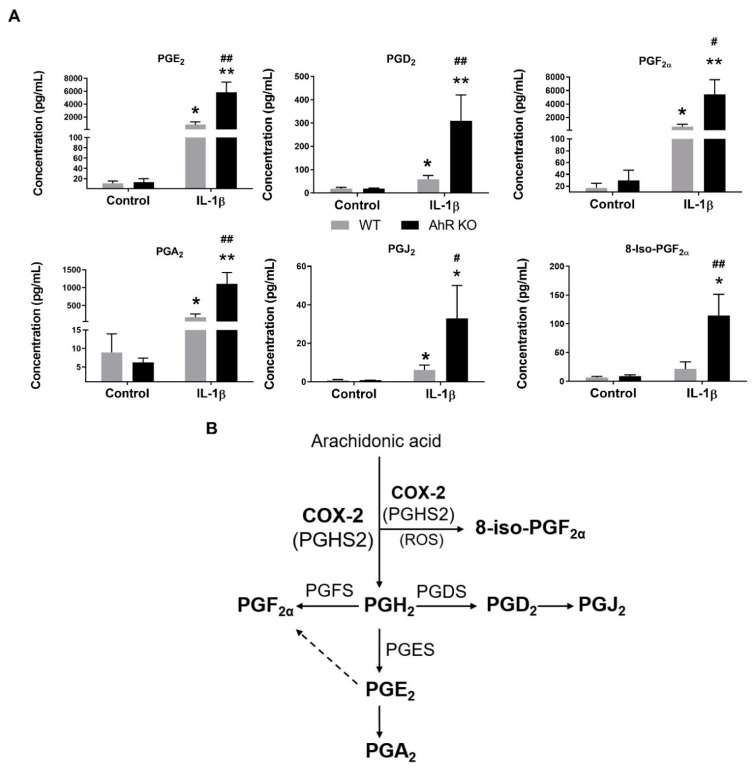
Loss of the AhR increases production of PGs in A549 cells exposed to model inflammatory stimulus. (**A**) A549 wild type (WT) and A549 AhR KO cells were exposed to IL-1β (10 ng/mL) or its diluent (negative control) for 24 h. Following the incubation, the concentrations of PGs in culture medium were assessed by LC-MS/MS. The data represent means + SD of four independent experiments. * and ** denote significant difference (*p* < 0.05 or *p* < 0.01, respectively) between the IL-1β-treated group and the respective control group. # and ## denote significant difference (*p* < 0.05 or *p* < 0.01, respectively) between the IL-1β-treated AhR KO cells group and the IL-1β-treated WT cells; (**B**) A schematic representation of the enzymatic production of PGs. PGs significantly upregulated in the AhR KO cells, treated with IL-1β, are highlighted in bold, including their unstable precursor PGH_2_. Possible formation of 8-iso-PGF_2α_ by oxidation induced by reactive oxygen species (ROS) is also indicated in the brackets.

PGs, generated from the released arachidonic acid (AA), are major regulators of inflammatory responses [30]. To evaluate the potential role of the AhR in the regulation of individual PG production in A549 cells, we exposed the AhR KO (clone H12) and the AhR WT cells to the pro-inflammatory cytokine IL-1β for 24 h, and we then determined the levels of eicosanoids in cell culture medium. As shown in Figure 2A, the concentrations of the secreted prostaglandins PGE_2_, PGD_2_, PGF_2__α_, PGA_2_, PGJ_2_ and 8-iso-PGF_2__α_ in cell culture medium collected from the AhR KO cells were significantly higher in comparison with the ones collected from the WT cells. The ratio of 8-iso-PGF_2α_ and PGF_2__α_ levels indicated that 8-iso-PGF_2__α_ had been primarily formed via enzymatic activity of prostaglandin-endoperoxide synthases, including COX-2 [31]. The list of analyzed eicosanoid species is provided in Supplementary Table S2. There were no significant differences between AA levels in the AhR KO and AhR WT cells, which suggests that the observed differences in PGs levels were not due to increased AA release. Interestingly, the levels of several lipoxygenase/cytochrome P450 products of AA metabolism [32] were significantly lower in the AhR KO cells (Supplementary Table S2). The downregulated eicosanoids included several hydroxyeicosatrienoic acids (HETE), in particular 11-HETE and 12-HETE, as well as 13-hydroxyoctadecadienoic acid, which is formed by several pathways both from AA and linoleic acid. Together, these results indicated that AA was preferentially utilized by COX-2, forming increasing levels of several PGs.

### 3.3. The AhR Disruption Increases Inducibility of COX-2 in A549 Cells 

**Figure 3:**
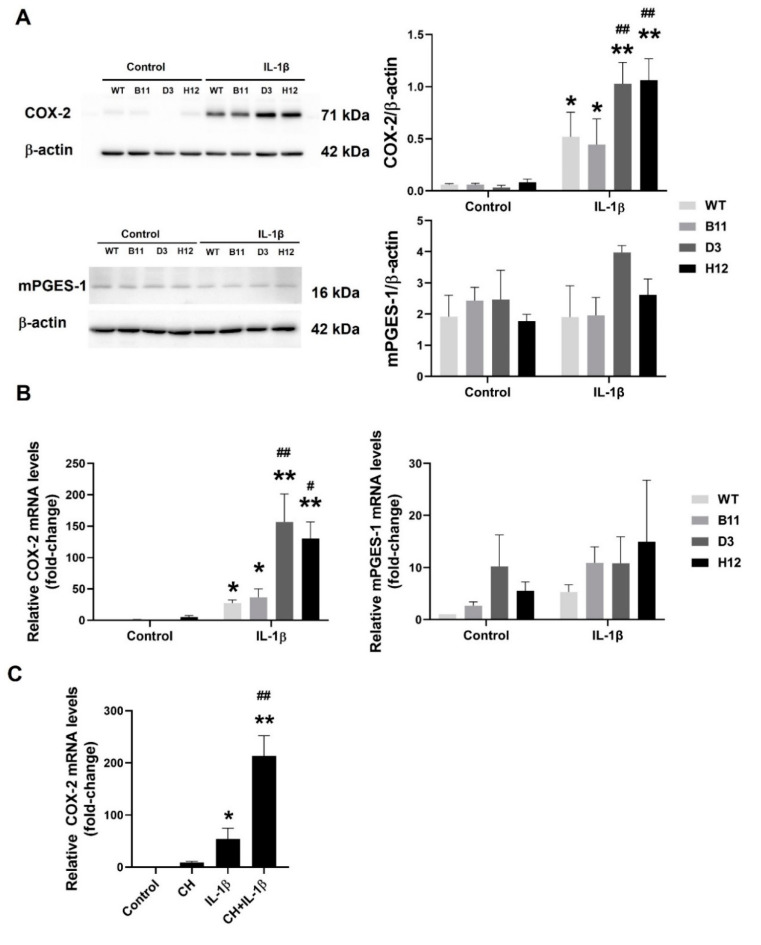
Loss of the AhR increases COX-2 inducibility in A549 cells. A549 WT, control B11 clone and the two A549 AhR KO clones (D3 and H12) were exposed to IL-1β (10 ng/mL) or its diluent (negative control) for 24 h. (**A**) After incubation, whole cell extracts were subjected to Western blotting analysis of COX-2 or mPGES-1 proteins. β-Actin was used as a loading control. The results are representative of three independent experiments. * and ** denote significant difference (*p* < 0.05 or *p* < 0.01, respectively) between the IL-1β-treated group and the respective control group. ## denotes significant difference (*p* < 0.01) between the respective IL-1β-treated AhR KO clone and the IL-1β-treated WT cells; (**B**) In parallel, total RNA was isolated and levels of COX-2 and mPGES-1 mRNAs were analyzed by RT-qPCR. The data are presented as means + SD of three independent experiments. * and ** denote significant difference (*p* < 0.05 or *p* < 0.01, respectively) between the IL-1β-treated group and the respective control group. # and ## denote significant difference (*p* < 0.05 or *p* < 0.01, respectively) between the respective IL-1β-treated AhR KO clone and the IL-1β-treated WT cells; (**C**) Effects of the AhR antagonist CH-223191 (CH) on induction of COX-2 mRNA induction by IL-1β in A549 cells. The A549 AhR WT cells were exposed to IL-1β (10 ng/mL), or its diluent (negative control) in the presence or absence of CH-223191 (10 μM; added 1 h before the treatment) for 24 h. Following the incubation, total RNA was isolated and COX-2 mRNA was quantified by RT-qPCR. The data represent means + SD of three independent experiments. * and ** denote significant difference (*p* < 0.05 or *p* < 0.01, respectively) between the IL-1β-treated group and the respective control group. ## Denotes significant difference (*p* < 0.01) between the IL-1β-treated cells and the cells treated with both IL-1β and CH-223191.

COX-2 is the main cyclooxygenase enzyme that is involved in production of a majority of PGs from AA [30,33,34]. Since we observed significantly higher levels of PGs being present in cell culture medium collected from the AhR KO cells, in particular PGE_2_ (Figure 2A), we next evaluated the induction of COX-2 and microsomal prostaglandin E synthase-1 (mPGES-1), which is responsible for PGE_2_ production, in the cells exposed to IL-1β. Both COX-2 protein (Figure 3A) and mRNA (Figure 3B) were significantly more upregulated by IL-1β in the A549 AhR KO cells than in the AhR WT cells. The increased induction of COX-2 protein and mRNA was observed in both the D3 and H12 clones, as compared with the WT cells, whereas in transfection control cells (B11), the levels of COX-2 were similar to the WT A549 cells. Microsomal PGES-1 is the primary enzyme responsible for the conversion of PGH_2_ to PGE_2_. However, the mPGES-1 mRNA levels were not significantly higher in the AhR KO cells, as compared with WT cells, thus indicating that the upregulation of COX-2 was responsible for the increased PGs production. 

Finally, in order to verify the effects of the AhR loss on COX-2 induction in A549 cells, we next employed also the AhR antagonist, CH-223191. As shown in Figure 3C, chemical inhibition of the AhR in AhR WT cells had a similar impact on COX-2 expression as full AhR KO: it significantly promoted the induction of COX-2 mRNA by IL-1β. 

### 3.4. Disruption of the AhR Signaling Leads to an Increased Production of Some Pro-Inflammatory Cytokines in A549 Cells

**Figure 4:**
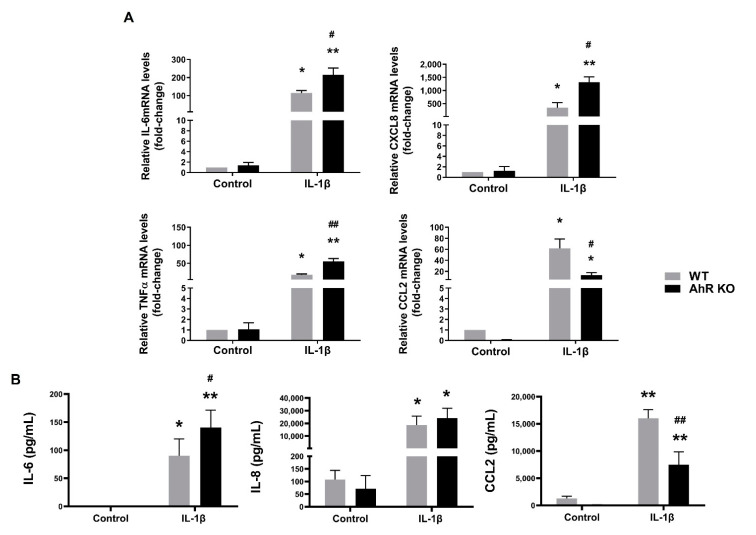
Disruption of the AhR signaling increases production of pro-inflammatory cytokines in A549 cells. (**A**) A549 WT and A549 AhR KO cells were exposed to IL-1β (10 ng/mL), or its diluent (negative control) for 24 h. Following the incubation, total RNA was isolated and the levels of IL-6, CXCL8, CCL2 and TNFα mRNAs were quantified by RT-qPCR; (**B**) A549 WT and A549 AhR KO cells were exposed to IL-1β (10 ng/mL), or its diluent (negative control) for 24 h. Following the incubation, the concentrations of IL-8, IL-6, and MCP1 (CCL2) in cell culture medium were measured by ELISA. The data are presented as means + SD of three independent experiments. * and ** denote significant difference (*p* < 0.05 or *p* < 0.01, respectively) between the IL-1β-treated group and the respective control group. # and ## denote significant difference (*p* < 0.05 or *p* < 0.01, respectively) between the respective IL-1β-treated AhR KO clone and the IL-1β-treated WT cells.

Next, we analyzed induction of expression of major pro-inflammatory cytokines (IL-6, CXCL8/IL-8 and TNFα) in cells exposed to IL-1β. As shown in Figure 4A, IL-6, CXCL8 and TNFα mRNAs were significantly higher in the AhR KO cells after IL-1β treatment, as compared with WT cells. We then measured the levels of cytokines in cell culture medium. Levels of IL-6 were significantly higher in cell culture medium collected from the AhR KO cells than in the AhR WT cells. We also assessed the production of the AhR-dependent chemokine (C-C motif) ligand 2 (CCL2/MCP-1) [35,36]. This cytokine has been shown to be directly regulated both by inflammatory signaling and by the AhR activity. Therefore, we expected that the impact of the AhR KO on its induction would be opposite than on induction of IL-6, CXCL8 or TNFα mRNAs. The IL-1β-induced levels of CCL2 mRNA, as well as CCL2/MCP-1 released from A549 cells into the cell culture medium, were lower in the A549 AhR KO cells than in the AhR WT cells, thus confirming that the AhR plays a direct role in the regulation of expression of this chemokine, unlike in the case of other evaluated pro-inflammatory cytokines (Figure 4). When using a chemical inhibitor of the AhR, CH-223191, we observed a trend towards increased IL-6 or CXCL8 levels in cells treated with both IL-1β and CH-223191; however, this was not significant. In contrast, induction of CCL2 mRNA was repressed in cells co-treated with CH-223191 (Supplementary Figure S2).

### 3.5. Disruption of the AhR Pathway Promotes NF-κB Activity in A549 Cells 

**Figure 5:**
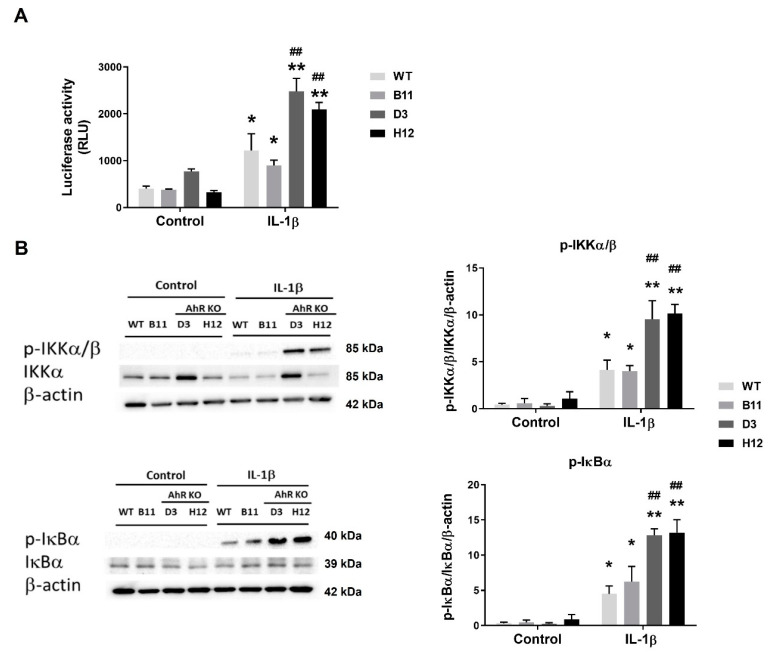
The AhR inhibits the NF-κB activity in A549 cells. (**A**) A549 AhR WT, A549 CRISPR/Cas9 empty vector (B11) and AhR KO D3 and H12 cells were transiently transfected with NF-κB luc reporter construct and pRL-TK vector encoding *Renilla* luciferase (transfection efficiency control). The transfected cells were exposed to IL-1β (10 ng/mL) or its diluent (negative control). Following the incubation, cells were collected, lysed and firefly/*Renilla* luciferase activities were determined; (**B**) A549 WT, B11, D3 and H12 cell variants were exposed to IL-1β (10 ng/mL) or its diluent (negative control) for 1 h (**left**) and 3 h (**right**). Following the incubation, whole cell extracts were subjected to immunoblot analysis of phosphorylated IKKα/β and IκBα. The images are representative of three independent experiments. The luciferase activity and densitometry data (expressed as phosphorylated protein relative to total protein and then normalized to β-actin levels) are presented as means + SD of three independent experiments. * and ** denote significant difference (*p* < 0.05 or *p* < 0.01, respectively) between the IL-1β-treated group and the respective control group. ## denotes significant difference (*p* < 0.01) between the IL-1β-treated AhR KO cells and the IL-1β-treated WT cells.

The above results have indicated that the AhR may act as a negative regulator of inflammation in A549 cells. The NF-κB pathway is one of the main pathways involved in the regulation of immunity, and it plays a major role in the control of inflammation [37]. The NF-κB signaling pathway can be activated by numerous pro-inflammatory molecules, including pro-inflammatory cytokines, reactive oxygen species, bacteria components, stress inducers, and a large array of drugs [38,39,40,41]. Since NF-κB signaling is reportedly affected by the AhR activity, we assessed the IL-1β-dependent increases in NF-κB-regulated reporter gene activity in the transiently transfected AhR WT or the AhR KO A549 cells. As shown in Figure 5A, IL-1β increased luciferase activity in both the WT and AhR KO cells; however, the reporter gene activity was significantly higher in the AhR KO cells.

To further analyze the possible negative regulatory role of the AhR in NF-κB signaling, we then determined the active state of the principal upstream regulators of NF-κB activity in the A549 WT and AhR KO cells exposed to IL-1β by using antibodies detecting their respective phosphorylated forms. We found that levels of phosphorylated IKKα/β kinases and their target, inhibitor of NF-kB α (IκBα), were higher in the AhR KO cells, as compared with the AhR WT cells (Figure 5B). Although there were some differences observed between the two AhR KO clones, D3 and H12, in total IKKα protein levels, both the levels of phosphorylated IKKα/β, and the activity of NF-κB-regulated reporter gene, were similar in the D3 and H12 AhR KO clones, thus suggesting that activation of NF-κB was similarly affected by the loss of the AhR. Taken together, the present results suggested that the AhR acts as a negative regulator of the NF-kB pathway activity in A549 cells in response to inflammatory stimuli.

### 3.6. DEP Extract and Its Polar Fraction Induce Exacerbated Inflammatory Response in A549 AhR KO Cells 

**Figure 6:**
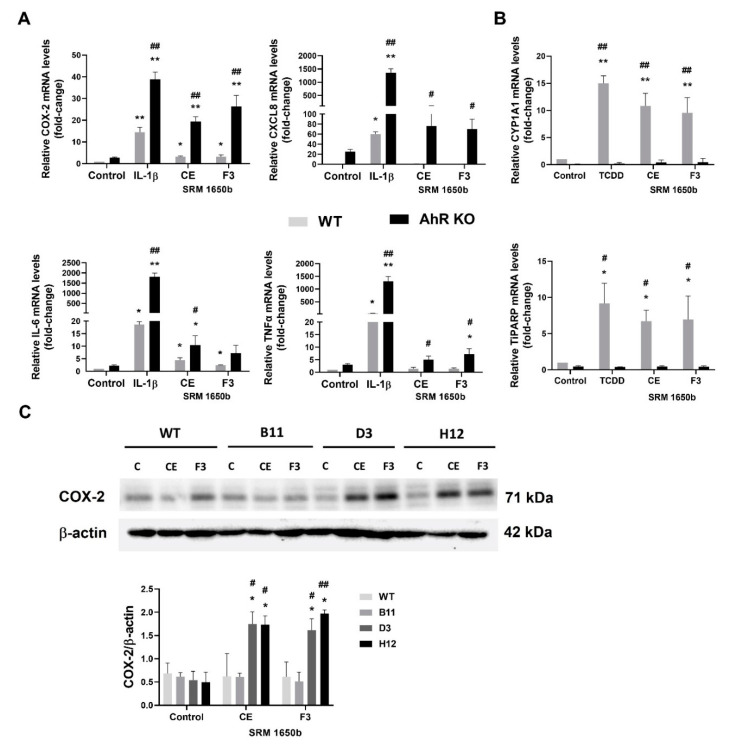
The AhR loss increases the inflammatory response of A549 exposed to a model mixture of airborne organic contaminants. A549 WT and A549 AhR KO cells (clone H12) were exposed to CE and polar F3 fraction of SRM 1650b at non-cytotoxic concentration (0.1 mg SRM equivalent/mL), or to DMSO (0.1% *v*/*v*; negative control) for 24 h. (**A**) Following the incubation, total RNA was isolated, and COX-2, TNFα, CXCL8 and IL-6 mRNAs were quantified by RT-qPCR. The data are presented as means + SD of three independent experiments; (**B**) Quantification of the AhR gene targets, CYP1A1 and TiPARP mRNAs in the A549 WT and A549 AhR KO cells (clone H12) exposed CE, F3 fraction or TCDD (10 nM, positive control). by RT-qPCR. The data are presented as means + SD of three independent experiments; (**C**) A549 WT, B11, D3 and H12 cell variants were exposed to CE, polar F3 fraction or DMSO (0.1% *v*/*v*; negative control) for 24 h. Following the incubation, whole cell extracts were subjected to immunoblot analysis of COX-2 levels. The image is representative of three independent experiments. The densitometry data (expressed as COX-2 levels normalized to β-actin levels) are presented as means + SD of three independent experiments. * and ** denote significant difference (*p* < 0.05 or *p* < 0.01, respectively) between the IL-1β-, CE-, F3- or TCDD-treated group and the respective control group. # and ## denote significant difference (*p* < 0.05 or *p* < 0.01, respectively) between the AhR KO cells and the WT exposed to the same treatment.

As discussed above, the lung epithelium is the first line of defense against particulate pollution and associated organic contaminants. Induction of aberrant inflammation is an important part of their toxic effects, and lipophilic organic chemicals bound to DEP have been shown to contribute to inflammatory responses in various types of target cells within the respiratory tract [25,42,43,44]. Therefore, we next aimed to characterize the role of the AhR after exposure to model airborne mixture of pollutants that activate inflammatory responses, by using crude organic extract of reference DEP mixture SRM1650b (CE) and its polar fraction (F3), which contains also a large number of polar derivatives of polycyclic aromatic compounds. These mixtures have been extensively characterized in our previous studies, and both CE and F3 have been shown to contain significant amounts of the AhR ligands [45,46]. As shown in Figure 6A, both CE and F3 fractions induced a significant inflammatory response in the AhR KO cells, which included the induction of COX-2, TNFα, CXCL8 and IL-6 mRNAs. The effects of CE and F3 fractions also included induction of the AhR gene targets, CYP1A1 and TiPARP, which were observed at the same concentration of CE and F3, respectively. Finally, both CE and F3 fraction were found to induce COX-2 protein in the AhR KO clones, but not in control (B11) or in the WT A549 cells (Figure 6C).

## 4. Discussion

Various types of combustion-derived airborne particles have been shown to interfere with signaling molecules involved in the regulation of immune responses within the lung tissue or in antioxidant defense of alveolar cells, including NF-κB and/or nuclear factor erythroid 2-related factor 2 (Nrf2) [47]. PAH mixtures, in particular those associated with airborne particulate matter (PM) or DEP (as well as other products of incomplete combustion processes), have been documented to induce production and/or release of various types of inflammatory mediators [25,43,44,48,49,50]. This exposes lung cells simultaneously to both inflammatory mediators upon PM exposure, and to the PM-associated AhR ligands (PAHs and related PAH derivatives, including alkylated, nitrated, or oxygenated PAHs). Both the AhR and inflammatory signaling pathways have been previously reported to interact in production of a variety of cytokines and chemokines [51,52,53]. On the other hand, in some cells or tissues, an increased AhR activity may dampen the inflammatory responses. Therefore, the role of the AhR could be two-sided, in both cell- and inflammatory stimulus-dependent manner. In the lungs, ATII cells are a rich source of cytokines and chemokines, orchestrating immune cell migration into the peripheral lung [54,55]. Disruption of inflammation control in ATII cells by the AhR ligands could be an important factor modulating their impact on the lung tissue. Therefore, in the present study, we addressed the functional role of the AhR in control of inflammatory responses in a model of ATII-like cells, A549 lung adenocarcinoma cell line.

We found that the loss of the AhR in A549 significantly increased inducibility of COX-2 mRNA/protein, when the cells were treated with IL-1β. This is in accordance with a previous observation that loss of the AhR or its silencing increases induction of COX-2 in various types of lung cells, in particular in lung fibroblasts, when treated with cigarette smoke extract or with inflammatory mediators [12,20,21]. An increased COX-2 expression in response to cigarette smoke extract has been also observed by the same group in another model of the AhR KO A549 cells generated by zinc finger nucleases [21]. However, a comprehensive evaluation of the impact of loss of the AhR on eicosanoid metabolism in lung cells is currently not available. Here, we show that the AhR KO leads to up-regulated production of both PGE_2_ and PGF_2α_, but also other, less prominent PGs, including PGD_2_ and PGJ_2_. This is in contrast with reduced levels of products of lipoxygenase/cytochrome P450-dependent metabolism of AA, thus suggesting that loss of the AhR, associated with an increased induction of COX-2 leads to a shift towards the prostaglandin branch of AA metabolism. 

Our findings about the role of the AhR in the control of AA metabolism may have implications not only for inflammatory reaction within alveoli, but also for our understanding of the development of lung cancer. Up-regulation of COX-2 and enhanced PGE_2_ production has been linked with increased cancer cell proliferation [56], angiogenesis, invasion, and metastasis [57]. Nevertheless, effects of various eicosanoids on cancer cells are PG-specific, and while PGE_2_ (and PGF_2α_) can promote inflammation and cancer progression in various tissues, the effects of PGD_2_ or PGJ_2_ appear to be ambiguous, as they may even suppress tumor growth and/or inflammation [58]. In our study, levels of PGE_2_ + PGF_2α_ were considerably higher than those of other PGs, thus suggesting that these two were predominantly formed. Such effects might contribute to COX-2-dependent growth and/or survival, previously observed in lung cancer cells, including A549 lung adenocarcinoma cells [59]. Deregulation of PG metabolism could also contribute to multiple roles that the AhR has been proposed to play in lung cancer development and metastasis [60,61]. Nevertheless, the production of PGE_2_ has been shown to play beneficial roles within the lung, as the activation of prostanoid receptors can promote the production of surfactants, inhibit inflammatory reaction, or promote wound healing [62]. In particular, E prostanoid receptor 4 has been shown to contribute to the anti-inflammatory effects of PGE_2_ in the lung, or to the maintenance of the lung endothelial barrier [63,64]. Therefore, the up-regulated PGE_2_ production in the absence of the AhR might affect distinct lung cell populations in a cell-dependent manner. 

Similar to an increased production of PGs, we also observed an upregulation of IL-6,IL-8 and TNFα expression, as well as IL-6 production, in the AhR KO A549 cells. Altered production of cytokines has been proposed to contribute to an increased neutrophilia in AhR KO mice treated with cigarette smoke extract [17,22]. Like COX-2, expression of these cytokines is under transcriptional control of NF-κB, and NF-κB has been shown to be more strongly activated by TNFα in bronchial epithelial cells with diminished AhR levels [65]. Here, we found significantly higher levels of NF-κB activity in the A549 AhR KO cells that were treated with IL-1β, when compared with their wild-type counterparts. The loss of the AhR was connected with increased phosphorylation of IKKα/β and IκBα, two key regulators of canonical NF-κB signaling [38]. These results indicate that the impact of the AhR on inflammation in A549 cells would lie upstream of NF-κB complex control, and that it might not involve direct AhR interactions with subunits of this transcription factor. In addition, our preliminary data also indicate that the effects observed in the AhR KO A549 cells are not observed in ARNT-deficient A549 cells (data not shown), which may indicate that the impact of the AhR on inflammation control is not related to the AhR/ARNT dimer binding to DRE elements, as previously suggested [22]. 

Several alternative mechanisms have been proposed to contribute to increased levels of COX-2 in cells with a reduced AhR content, including its post-transcriptional regulation by HuR, which stabilizes COX-2 mRNA and thus promotes COX-2 mRNA translation [20], or the AhR interactions with various NF-κB subunits, including both RelA and RelB [12,14,15]. Our data indicate that the role of the AhR in A549 cell model would lie upstream of IκBα, and that they could be linked with the control of IKKα functions. Interestingly, IKKα may directly interact with the AhR and this cross-talk has been proposed to mediate chromatin remodeling, which might alter both the AhR-dependent transcription and expression of genes linked with stem-like properties of cancer cells [66,67]. It is possible that the AhR could sequester IKKα in wild-type cells, and thus alter the control of NF-κB signaling. Taken together, a combination of several mechanisms targeting NF-kB activation, described previously or identified in this study, may contribute to increased inflammatory responses in lung epithelial cells.

Exposure of lung cells to cigarette smoke, ambient PM, or other types of particles derived from incomplete combustion processes, including DEP, poses a serious threat to human health [1]. It may increase cancer risk, especially when combined with other known risk factors [68]. Numerous types of airborne particles also contribute to the risk of respiratory diseases and atherosclerosis, or further cardiovascular and reproductive effects [68,69,70]. The latter effects are often associated with stimulation of the production of pro-inflammatory mediators and oxidative stress [23,25,43,44,71,72]. This complex interplay of pro-oxidative and pro-inflammatory effects of particles themselves is likely to be modulated by potent AhR ligands that are adsorbed to the particles contributing to ambient and traffic-related air pollution. It has been shown that lack of the AhR increases pulmonary neutrophilia in mice exposed to cigarette smoke, which contains large quantities of both the AhR ligands and compounds inducing inflammation [22]. Here, using a defined mixture of organic pollutants derived from reference DEP material, we showed that both CE and its polar fraction are capable of inducing of exacerbated inflammatory response (including induction of COX-2 and inflammatory cytokines) in the AhR KO A549 cells as compared with WT cells. Shang et al. [73] have concluded that inflammatory effects of organic extracts of traffic-related PM could be related to the action of quinones and production of reactive oxygen species. This seems to be supported by our observation that polar F3 fraction (which is known to contain also significant amounts of quinones and other polar PAH derivatives) was responsible for the induction of COX-2 expression, since it induced similar levels of COX-2 mRNA, or COX-2 protein, as crude extract (CE) of SRM1650b DEP material in the AhR KO cells. This suggests that polar compounds induced COX-2 in an AhR-independent manner. Some effects of single PAHs, such as BaP, on COX-2 induction, e.g., in human bronchial BEAS-2B cells, have also been reported, but considered to be AhR independent [74]. Similarly, BaP did not induce chemokine response (including induction of CXCL8) in BEAS-2B cell line, unlike some nitrated PAHs, which seemed again to act in an AhR-independent manner [75]. It is possible, given the available evidence, that induction of COX-2 expression by some PAHs, or by their mixtures, in bronchial or lung epithelial cell models, is not mediated directly via the AhR action, but indirectly via the AhR -dependent metabolism of PAHs, which yields both ROS and active quinone PAH metabolites. Nevertheless, the present data should be interpreted with some caution. As our observations here are limited to A549 adenocarcinoma cells model, cultivated in a standard submerged culture mode, future studies should further address these mechanisms in vivo, or in more advanced models of ATII cells. In addition, it has been shown that the activation of the AhR simultaneously promotes oxidative stress and contributes to bioactivation of carcinogens [11,76]. Therefore, the AhR action may still promote further toxic events linked with lung disease, including cancer. Importantly, the role of the AhR in control of inflammation in vivo is certainly more complex [44], in particular due to the fact that the AhR is highly expressed in numerous immune cell types within the respiratory tract. It is known to directly regulate their functions and responses, including their responses to the AhR ligands found in polluted environments [42]. The multiple roles of the AhR in fine tuning the lung immune responses thus deserve more attention in future studies.

## 5. Conclusions

The AhR has been proposed to act as an important regulator of the immune system within the respiratory tract, and, it has been suggested, to impact various aspects of lung health [76,77]. The lungs are vulnerable to inhaled toxicants, and many of these organic pollutants are also efficient AhR agonists. Our present data indicate that the AhR plays largely a suppressive role in the control of inflammatory reaction in A549 cells (a widely used model of ATII-like cells), via inhibition of the NF-κB activity, which then reduces COX-2 expression/activity, alters eicosanoid metabolism, and decreases expression/production of several inflammatory cytokines and chemokines. Nevertheless, it should be noted that the AhR may also directly regulate the expression of several inflammatory mediators [42,78], and that its effects may also depend on the nature of inflammatory stimulus and immune cells being present within alveoli. The future studies should address the relevant mechanisms also in additional, non-cancer derived models of human ATII cells, as well as to evaluate their interactions with the immune cells. Such studies may help us to better understand inflammatory effects of airborne pollutants within lung epithelium.

## Data Availability

The data presented in this study are available on request from the corresponding authors.

## Supplementary Materials

The following are available online at https://www.mdpi.com/article/10.3390/cells11040707/s1: Figure S1: Sequencing data; Figure S2: Modulation of IL-6, IL-8 and CCL2 mRNA induction by CH-223191 in A549 AhR KO cells. Table S1: Primer and probe sequences used for RT-qPCR; Table S2: Eicosanoid quantification in A549 WT and AhR KO cells treated with IL-1β.
