# Supplementary material for: Aryl Hydrocarbon Receptor (AhR) Limits the Inflammatory Responses in Human Lung Adenocarcinoma A549 Cells via Interference with NF-κB Signaling
Source: Cells. 2022 Feb 17;11(4):707. doi: 10.3390/cells11040707 (PMC8870046; doi:10.3390/cells11040707)
Supplement: Supplementary file 1 [file cells-11-00707-s001.zip › cells-1497800-supplementary.pdf]

cells

Article

Aryl Hydrocarbon Receptor (AhR) Limits the Inflammatory Responses in Human Lung Adenocarcinoma A549 Cells via Interference with NF-κB Signaling

Gerardo Vázquez-Gómez, Martina Karasová, Zuzana Tylichová, Markéta Kabátková, Aleš Hampl, Jason Matthews, Jiří Neča, Miroslav Ciganek, Miroslav Machala and Jan Vondráček

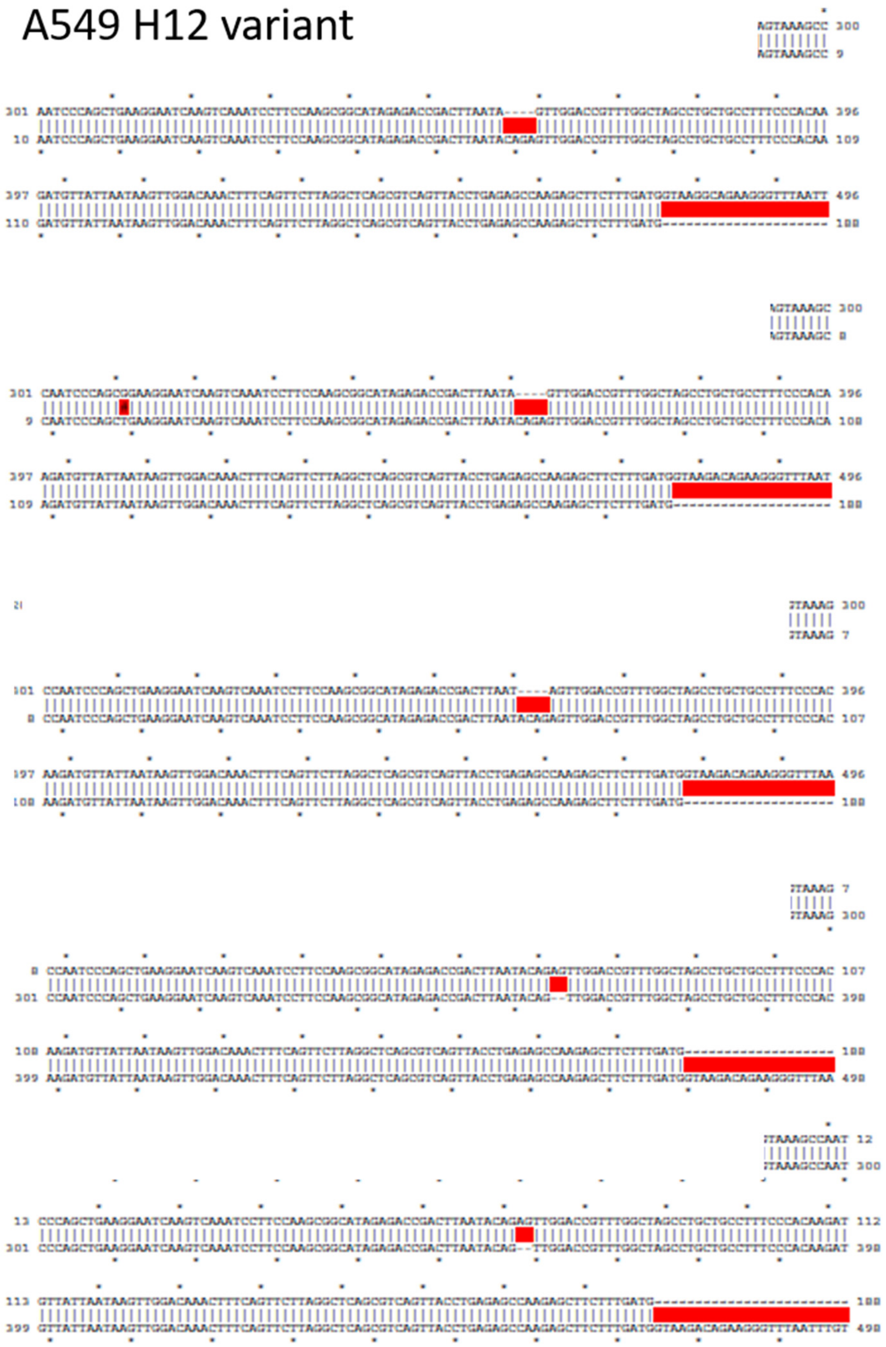

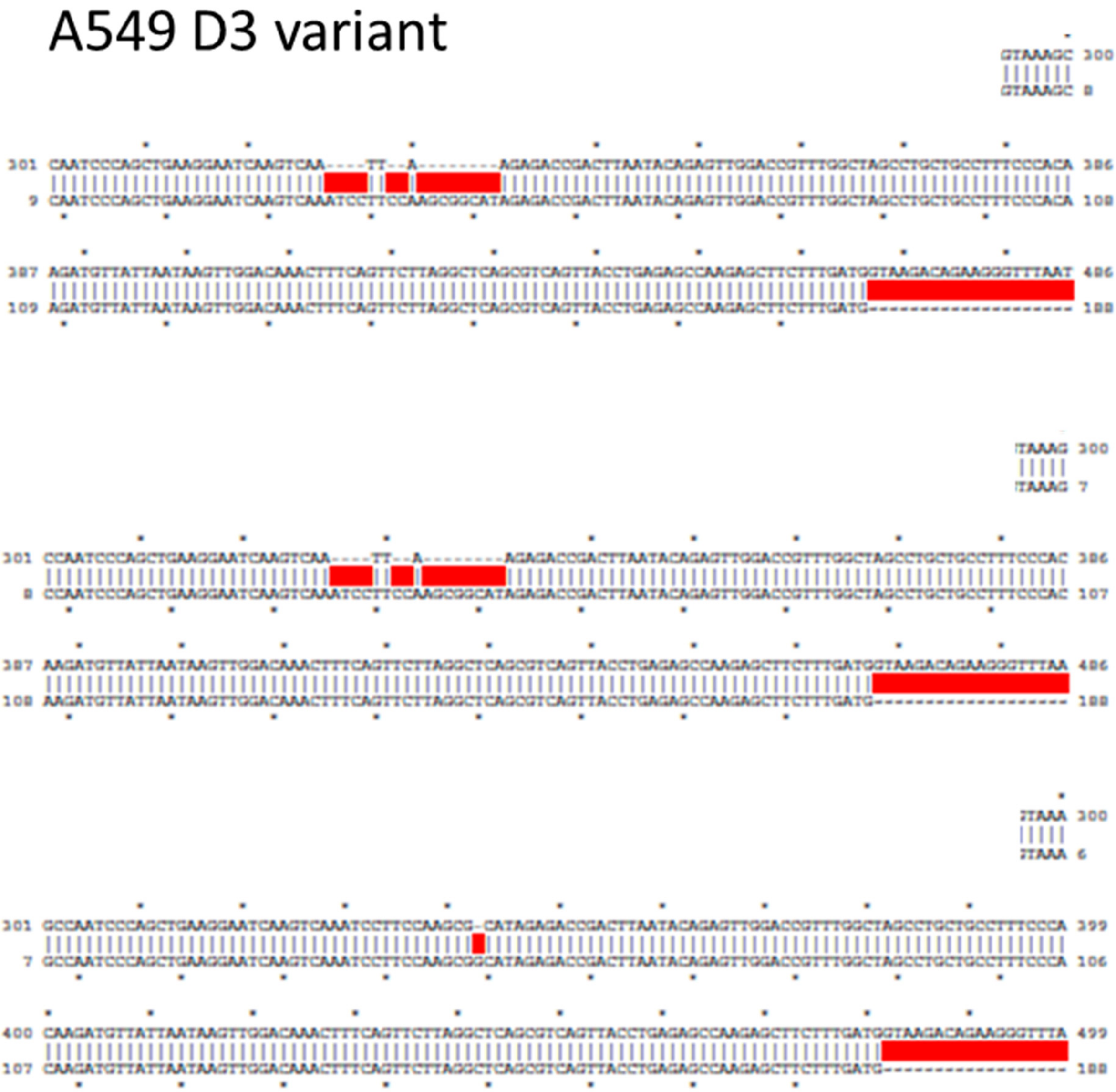

Figure S1. Sequencing data shown as alignment of tested sequences bearing mutations (deletions) and reference sequence AhR gene (exon 2). Clone H12 contains deletions CAGA and AG, both causing frameshift in reading frame. Clone D3 contains longer deletion ATCC-CC-AGCGGCAT and 1 nt deletion of G. Presence of two different mutations confirms bi-allelic knockout of AhR gene.

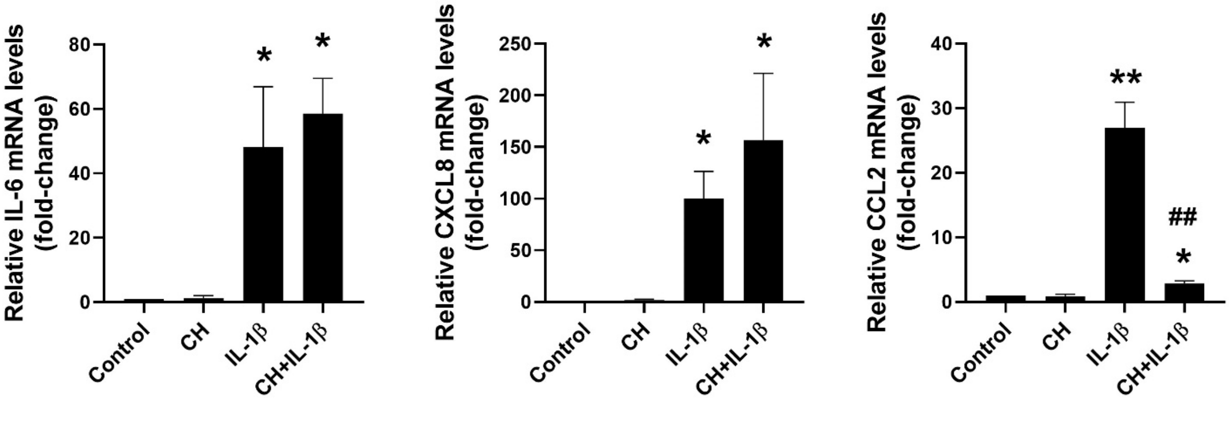

Figure S2. Effects of AhR antagonist CH-223191 on induction of IL-6, IL-8 and CCL2 mRNAs induction by IL-1β in A549 cells. A549 AhR WT cells were exposed to IL-1β (10 ng/mL), or its diluent (negative control) in presence or absence of CH-223191 (10 μM; added 1 h before the treatment) for 24 h. Following the incubation, total RNA was isolated and IL-6, CXCL8 and CCL2 mRNAs were quantified by RT-qPCR. The data represent means + SD of three independent experiments. * and ** denote significant difference ($P<0.05$ or $P<0.01$, respectively) between the IL-1β-treated group and the respective control group. ## Denotes significant difference ($P<0.01$) between the IL-1β-treated cells and the cells treated simultaneously with IL-1β and CH-223191.

Table S1. Primer and probe sequences used for RT-qPCR.

Gene/accession no.	Primer/probe	Primer and probe sequences
CYP1A1<br>NM_000499.3	Fw.<br>Rev.<br>Probe	5′-CACCATCCCCCACAGCAC-3′<br>5′-TTACAAAGACACAACGCCCC-3′<br>5′-CAAGTTTGAAAGGCTTTTACATCCCC-3′
IL-6<br>NM_000600.4	Fw.<br>Rev.<br>Probe	5′-GATGAGTACAAAAGTCCTGATCC-3′<br>5′-CTGCAGCCACTGGTTCTGT-3′<br>UPL # 40
CXCL8<br>NM_000584.38	Fw.<br>Rev.<br>Probe	5′-AGACAGCAGAGCACACAAGC-3′<br>5′-ATGGTTCCTTCCGGTGGT-3′<br>UPL #72
CCL2<br>NM_002982.3	Fw.<br>Rev.<br>Probe	5′-AGTCTCTGCCGCCCTTCT-3′<br>5′-GTGACTGGGGCATTGATTG-3′<br>UPL #40
TNF<br>NM_000594.3	Fw<br>Rev.<br>Probe	5′-CAGCCTCTTCTCCTTCCTGAT-3′<br>5′-GCCAGAGGGCTGATTAGAGA-3′<br>UPL #29
mPGES-1<br>NM_004878.4	Fw.<br>Rev.<br>Probe	5′-CCCTGAGTCCTGGTTTCCT-3′<br>5′-CACACACACAGGCCCACT-3′<br>UPL# 40
TBP (ref. gene)<br>NM_003194.4	Fw.<br>Rev.<br>Probe	5′-GAACATCATGGATCAGAACAACA-3′<br>5′-ATAGGGATTCCGGGAGTCAT-3′<br>UPL #87
COX-2<br>NM_000963.2	Fw.<br>Rev.<br>Probe	5′-CTTCACGCATCAGTTTTTCAAG-3′<br>5′-TCACCGTAAATATGATTTAAGTCCAC-3′<br>UPL #23
TIPARP<br>NM_001184717	Fw.<br>Rev.<br>Probe	5′-GGAAATTCTTCTGTAGGGACCA-3′<br>5′-AATCAATCGAATGACAGACTCG-3′<br>UPL #58
IL-1B<br>NM-000576.2		TaqMan® Assay Probe ID: Hs01555410_m1.<br>Thermo Fisher, Cat. No. 4331182

Table S2. Eicosanoid quantification in A549 WT and AhR KO cells treated with IL-Iβ. Significant differences between AhR WT cells and AhR KO cells are highlighted in bold.

Eicosanoid	WT Control		AhR KO Control			WT IL-1β		AhR KO IL-1β		
	Mean	SD	Mean	SD	*p*-value	Mean	SD	Mean	SD	*p*-value
Arachidonic acid [a]	34.2	8.4	21.1	3.3	0.2313	43.5	11.9	26.5	5.1	0.0878
PGE_2	11.1	4.2	13.2	6.1	>0.9999	**811.7**	379.5	**5820.8**	1370.4	<0.0001
PGA_2	8.9	4.4	6.2	1.0	>0.9999	**171.5**	77.6	**1100.5**	278.7	<0.0001
PGD_2	18.1	5.4	17.7	3.3	>0.9999	**59.5**	13.6	**310.3**	96.0	0.0002
PGJ_2	0.9	0.3	0.8	0.1	>0.9999	**6.1**	2.3	**32.9**	14.8	0.0043
$PGF_{2\alpha}$	17.4	6.7	29.9	15.0	>0.9999	**630.9**	315.1	**5434.2**	1880.9	0.0002
8-Iso-$PGF_{2\alpha}$	6.4	1.7	8.7	2.0	>0.9999	**21.2**	10.8	**114.8**	31.6	0.0001
$PGF_{2\beta}$	32.7	17.6	19.0	5.3	0.9972	345.5	155.8	178.3	68.9	0.1318
15-keto-PGE_2	2.9	0.6	2.0	0.6	0.9618	4.0	1.8	7.2	4.2	0.3803
13,14-DH-15-keto-PGE_2	9.7	4.4	4.3	1.7	>0.9999	775.8	360.4	975.2	273.8	0.8218
13,14-DH-15-keto-PGD_2	1.3	0.1	1.5	0.2	0.9947	8.1	1.6	8.0	2.2	0.9991
20-HETE	25.5	9.8	22.1	9.7	0.9856	35.3	18.8	25.2	7.6	0.7493
13-HODE	57.6	14.0	36.7	12.0	0.3365	**122.0**	17.8	**41.5**	13.7	0.0001
9-HODE	32.0	5.8	19.9	13.1	0.296	34.1	5.7	22.3	4.0	0.315
15-HETE	18.3	5.6	7.2	2.2	0.0806	16.8	7.6	8.1	2.8	0.1999
11-HETE	140.1	63.1	31.3	7.6	0.0898	**247.8**	71.9	**87.9**	33.2	0.0106
8-HETE	**31.7**	12.8	**4.8**	2.5	0.0052	20.7	7.3	6.5	3.5	0.164
12-HETE	**119.6**	13.9	**11.5**	3.6	<0.0001	**114.3**	20.2	**14.1**	7.0	<0.0001
9-HETE	4.5	3.1	1.9	0.3	0.4615	4.4	3.0	2.0	0.5	0.5377
5-HETE	17.2	5.7	9.3	4.1	0.1675	15.3	4.6	9.1	2.3	0.342

[a] The levels of AA are shown in ng/ml. All other values are in pg/ml of cell culture medium.
